# Survival benefits and prognostic models of surgical resection versus continued medical treatment in CNLC stage IIb/IIIa hepatocellular carcinoma after meeting resectability criteria following conversion therapy

**DOI:** 10.3389/fonc.2026.1958780

**Published:** 2026-10-14

**Authors:** Jian Qiu, Zhenghui Ye, Liyang Zhao, Wei Liu, Shenao Liu, Hongchuan Zhao

**Affiliations:** Department of Hepatobiliary, Pancreatic, and Transplantation Surgery, The First Affiliated Hospital of Anhui Medical University, Hefei, China

**Keywords:** conversion therapy, hepatocellular carcinoma, medical treatment, resectability criteria, surgical resection

## Abstract

**Objective:**

The combination of targeted therapy, immunotherapy, and locoregional treatment has established conversion therapy as a viable strategy for China Liver Cancer Staging (CNLC) stage IIb/IIIa hepatocellular carcinoma (HCC). However, upon meeting the criteria for tumor resectability after successful conversion, it remains controversial whether surgical resection (SR) provides superior survival benefits compared to continued medical treatment (CMT), and the optimal timing for surgery is still undefined.

**Methods:**

We retrospectively analyzed 363 CNLC stage IIb/IIIa HCC patients who met strict tumor resectability criteria after a conversion regimen of lenvatinib, anti-PD-1 antibodies, and transarterial chemoembolization (TACE). Patients were randomly assigned to a training cohort (n = 255) and an internal validation cohort (n = 108) at a 7:3 ratio. Propensity score matching (PSM) was utilized to balance the baseline characteristics. Survival outcomes were compared and prognostic nomograms were developed using Cox proportional hazards regression.

**Results:**

After 1:3 PSM, the training cohort comprised 63 SR patients and 189 CMT patients. The SR group demonstrated significantly better median overall survival (mOS, 32.1 vs. 24.3 months; P < 0.0001) and median progression-free survival (mPFS, 15.3 vs. 10.6 months; P = 0.0041) than the CMT group. The prognostic nomograms developed for both groups exhibited excellent discrimination via bootstrap resampling and in the internal validation cohort. Restricted cubic spline (RCS) and Kaplan-Meier analyses revealed that an interval > 90 days from meeting resectability criteria to surgery, and a pre-operative AFP to baseline AFP (at the time resectability was met) ratio > 1, were significantly associated with worse OS.

**Conclusion:**

For CNLC stage IIb/IIIa HCC patients achieving tumor resectability following conversion therapy, SR is significantly associated with improved survival outcomes compared to CMT. Based on our exploratory findings, surgery within 90 days of meeting resectability criteria may be considered a beneficial strategy, provided that the pre-operative AFP level does not exceed the level observed when resectability was initially achieved.

## 1 Introduction

Hepatocellular carcinoma (HCC) is one of the leading causes of cancer-related mortality globally (1, 2). In China, a substantial proportion of patients present with intermediate-to-advanced disease at initial diagnosis (3–5). According to the 2026 Chinese Guidelines for the Diagnosis and Treatment of Primary Liver Cancer, systemic therapy—particularly the combination of tyrosine kinase inhibitors (TKIs) and immune checkpoint inhibitors (ICIs)—coupled with locoregional therapy is the highly recommended conversion strategy for CNLC stage IIb/IIIa HCC (6). The rapid evolution of these multimodal interventions has profoundly reshaped the HCC treatment landscape (7–10), significantly enhancing objective response rates and successfully downstaging a considerable number of initially unresectable patients to meet surgical criteria (2, 11–13).

“Tumor resectability criteria” represent a rigorous surgical evaluation framework. The 2023 JLCA/JSHBPS expert consensus defines resectability based on tumor number, diameter, vascular invasion, and distant metastasis (14). For Chinese patients, Ma Z et al. proposed resectability criteria following conversion for CNLC stage IIb/IIIa HCC, which include significant decreases in 1) anticipated R0 resection; 2) preservation of ≥2 contiguous liver segments with intact vascular inflow/outflow and biliary drainage; 3) ECOG PS 0–1; 4) Child-Pugh class A or B; 5) ICG-R15 <30%; and 6) adequate future liver remnant (FLR >30% for normal livers; >40% for cirrhosis) (15). According to the Chinese Expert Consensus on Conversion Therapy for Hepatocellular Carcinoma (2021 Edition), oncological resectability for stage IIb-IIIa HCC requires downstaging with MVTT necrosis (mRECIST criteria), partial response, or stable disease maintained for 3 to 4 months (12). This study adopted these 2021 Edition rigorous criteria for patient selection.

Prolonged OS and PFS remain the ultimate goals of HCC therapy. Although systemic treatments can induce deep or sustained remissions, most responders maintained solely on medical therapy eventually experience disease progression within 1.0 to 1.5 years (11, 12, 16–19). Consequently, clinicians face a critical clinical dilemma for patients achieving profound responses: whether to aggressively pursue SR or CMT.Furthermore, for patients opting for SR, determining the optimal surgical window remains a major challenge. Relying strictly on imaging is risky, as radiological changes often lag behind the development of acquired immune tolerance, potentially missing the opportunity for curative resection (17, 20–22). Comprehensive metrics and a well-defined time frame—calculated from the exact moment resectability criteria are met—are therefore urgently needed (2, 23).To address these clinical challenges, this study aims to compare survival outcomes between SR and CMT strictly in CNLC stage IIb/IIIa HCC patients meeting resectability criteria, construct predictive prognostic models, and explore a potential time window for surgical intervention.

## 2 Methods

### 2.1 Data collection

We retrospectively collected data from patients initially diagnosed with CNLC stage IIb/IIIa HCC who received a conversion regimen consisting of lenvatinib, anti-PD-1 antibodies and TACE at the First Affiliated Hospital of Anhui Medical University between January 2022 and December 2025. Inclusion criteria were (1): initial diagnosis of CNLC stage IIb/IIIa (2); treatment with the aforementioned triple therapy (3); objective achievement of tumor resectability criteria during the disease course, as confirmed by our retrospective review of clinical and radiological data. Exclusion criteria included (1): failure to complete the scheduled treatment courses (2); prior treatments such as hepatectomy, radiofrequency ablation (RFA), TACE, or other systemic therapies (3); concurrent use of other antineoplastic therapies (e.g., hepatic arterial infusion chemotherapy, radiotherapy) (4); presence of other malignancies; and (5) incomplete clinical data or loss to follow-up. The final enrolled patients were randomly allocated to a training cohort (n = 255) and an internal validation cohort (n = 108) at a 7:3 ratio.

The study protocol was approved by the Clinical Research Ethics Committee of the First Affiliated Hospital of Anhui Medical University and was performed in accordance with the ethical guidelines of the 1975 Declaration of Helsinki (ethical number: 2022423). Written informed consent was obtained from all patients.

For data annotation, baseline laboratory parameters prior to conversion therapy were suffixed with “_1”, those measured upon meeting resectability criteria with “_2”, and preoperative measurements in the SR group with “_3”. The interval from initiating conversion therapy to meeting resectability criteria was recorded in months, while the interval from achieving resectability to surgery was recorded in days. Survival times (OS and PFS) were calculated in months. The variable “preoperative AFP to resectability baseline AFP ratio” was treated as a binary status (assigned 1 if > 1, and 0 if ≤ 1).

### 2.2 Treatment regimen

Lenvatinib was administered orally once daily (8 mg for body weight < 60 kg, and 12 mg for weight ≥ 60 kg), combined with intravenous anti-PD-1 antibodies (camrelizumab 200 mg, sintilimab 200 mg, tislelizumab 200 mg, or pembrolizumab 200 mg) every 3 weeks (24, 25). Super-selective TACE was performed under local anesthesia via a femoral artery approach (typically right-sided). Initial angiography of the celiac trunk and/or common hepatic artery was performed to delineate tumor number, size, location, feeding arteries, and anatomical variations. Selective mesenteric arteriography was added as needed to identify accessory supply.​ An emulsion of lipiodol (dose tailored to tumor burden and liver function, typically 5–20 mL) and pirarubicin was administered super-selectively via a microcatheter. To mitigate the risk of nontarget embolization and liver failure, lipiodol was avoided in cases of significant arterioportal shunting.​ Subsequent embolization with gelatin sponge particles was continued until near-stasis of antegrade flow​ was achieved (26, 27). Repeat TACE sessions were scheduled at 4- to 6-week intervals based on contrast-enhanced CT or MRI, performed according to modified Response Evaluation Criteria in Solid Tumors (mRECIST) criteria (28), if viable tumor with persistent arterial enhancement was identified.​ Refractoriness to two consecutive TACE sessions prompted consideration of alternative therapies and exclusion of the patient from this study. All patients co-infected with the hepatitis B virus (HBV) received oral antiviral agents (entecavir or tenofovir) (20).

Patients were systematically followed up at least every 3 months. Hepatic function, treatment-related adverse events (TRAEs) (29), tumor response, and resectability were evaluated utilizing clinical, laboratory, and radiological data. Tumor responses were determined according to the mRECIST. For Grade 3 or higher TRAEs, systemic therapies were temporarily suspended until recovery to Grade 1 or baseline, followed by a stepwise dose reduction for lenvatinib (e.g., from 12 mg to 8 mg).

### 2.3 Retrospective resectability assessment and grouping

An independent multidisciplinary team (MDT)—comprising medical oncologists, infectious disease specialists, hepatobiliary surgeons, radiologists, and interventional radiologists—conducted a retrospective centralized review of the clinical, radiological, and laboratory data. In cases of disagreement on resectability, a final decision was reached by a majority vote requiring at least two-thirds agreement among the MDT members. This review aimed to identify patients within the real-world cohort who strictly met the aforementioned “tumor resectability criteria” during their disease course. Among the patients confirmed to have met these criteria retrospectively, they were categorized into the SR group or the CMT group based on the actual clinical interventions they received at the time. Patients in the SR group underwent partial hepatectomy after providing informed consent to their primary care teams.

### 2.4 Study endpoints

The primary endpoints were OS (defined as the time to patient death from any cause or the study cutoff date) and PFS (defined as the time to disease recurrence/progression or the study cutoff date). The starting point for calculating both OS and PFS was standardized as the exact date the patient first met the tumor resectability criteria. The final follow-up cutoff date was December 31, 2025.

### 2.5 Statistical analysis

To mitigate selection bias, a 1:3 propensity score matching (PSM) was performed. The propensity score was estimated using a multivariable logistic regression model that included the following baseline covariates: age, gender, Child-Pugh class, ECOG PS, and CNLC stage. Matching was executed using the nearest-neighbor matching algorithm without replacement, with a caliper width set at 0.2 of the standard deviation of the logit of the propensity score. The adequacy of the matching was strictly evaluated using the standardized mean difference (SMD). An SMD < 0.1 for all baseline covariates was considered indicative of optimal group balance. Continuous variables were expressed as mean ± standard deviation for normally distributed data, or median and interquartile ranges (IQR) for skewed distributions. Categorical variables were presented as counts and percentages and compared using Chi-square tests. Survival curves (OS and PFS) were estimated by the Kaplan-Meier method and compared using the Log-rank test. Univariate and multivariate Cox proportional hazards models were utilized to identify independent prognostic factors, which were then used to formulate predictive nomograms. The accuracy and clinical utility of these models were evaluated using time-dependent receiver operating characteristic (ROC) curves, calibration plots, and decision curve analysis (DCA). Restricted cubic splines (RCS) were employed to explore potential non-linear relationships between the interval to surgery and the risk of mortality. To facilitate clinical application and point-of-care decision-making, an interactive web-based calculator was developed for both the SR group and the CMT group. The online tool allows users to select the treatment modality, input individual baseline parameters (such as CNLC stage, AFP ratio status, and interval to surgery), and specify the prediction timeframe. The system dynamically computes the individualized overall survival (OS) probability and generates the corresponding predicted survival curve. Model optimism and stability during development were evaluated using bootstrap resampling (1, 000 replicates) in the training cohort, and model performance was subsequently evaluated in the internal validation cohort.The application is publicly accessible at https://jianqiu.shinyapps.io/hcc-predictor/. All statistical analyses were executed utilizing R software (version 4.4.3), and a two-sided P < 0.05 was considered statistically significant.

## 3 Results

### 3.1 Baseline characteristics and safety outcomes after PSM

Among the 860 CNLC stage IIb/IIIa patients treated with the triple conversion regimen, 635 (73.84%) achieved initial disease control, yet only 363 (42.21%) satisfied the criteria for tumor resectability. These 363 patients were randomized 7:3 into a training cohort (n = 255) and an internal validation cohort (n = 108). Within the training cohort, 63 patients underwent SR and 192 continued CMT based on the study design criteria. After 1:3 PSM, the final analysis matched 63 SR patients with 189 CMT patients (shown in **Figure 1**). Baseline characteristics were well-balanced post-matching (P > 0.05, SMD < 0.1, **Table 1**). Regarding safety profiles, the SR group exhibited a significantly lower incidence of grade 1–2 TRAEs compared to the CMT group (41.27% vs. 62.96%, P = 0.004). The incidences of grade 3 TRAEs were comparable (3.17% vs. 5.29%, P = 0.733), and no grade 4 or higher TRAEs were recorded in either group.

**Figure 1:**
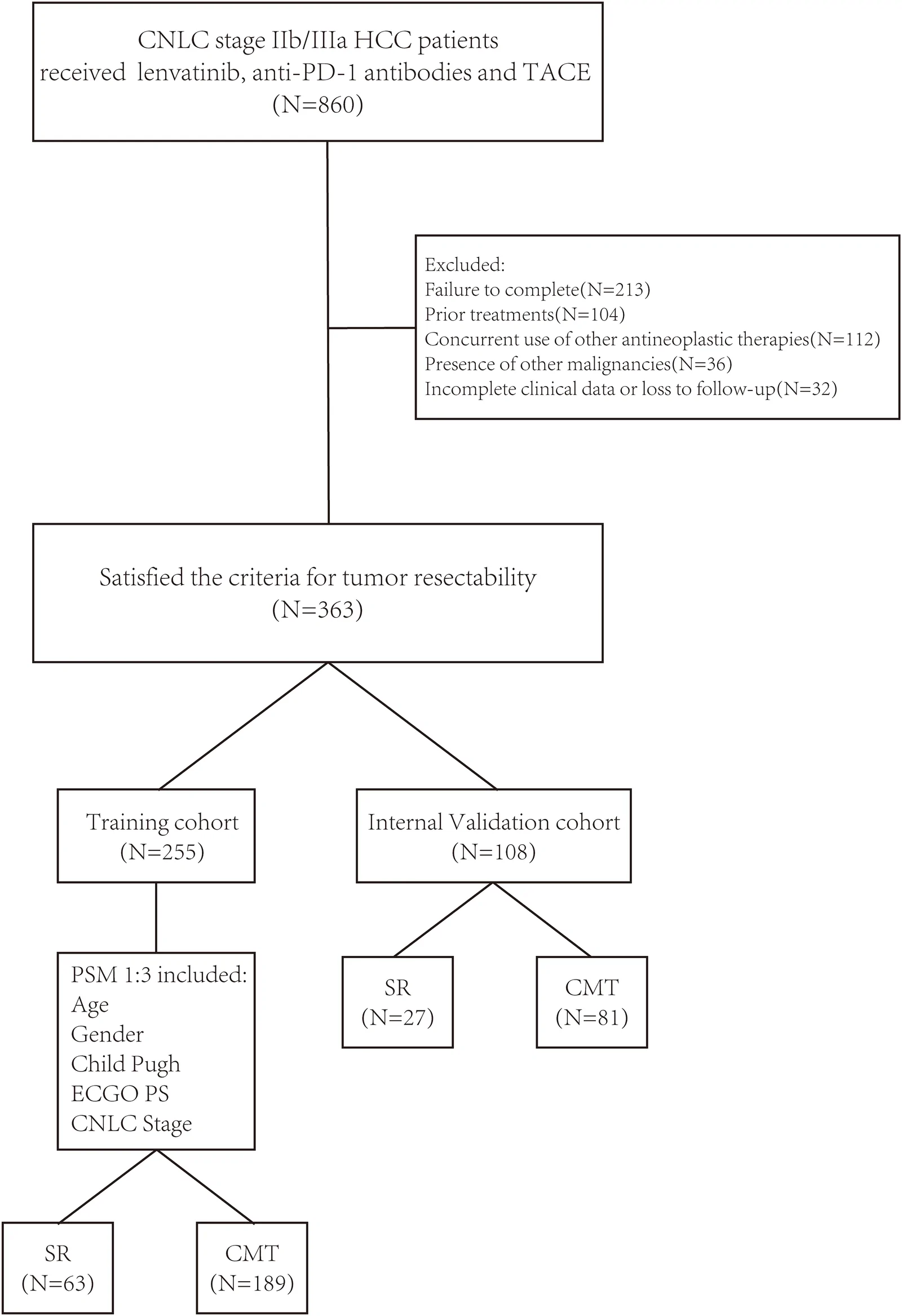
Flowchart of patients selection. CNLC, China liver cancer; HCC, Hepatocellular carcinoma; TACE, Transarterial chemoembolization; PSM, Propensity score matching; ECOG PS, Eastern cooperative oncology group performance status; SR, Surgical resection; CMT, Continued medical treatment.

**Table 1:** Baseline characteristics after PSM: SR vs. CMT​.

Variables	SR	CMT	*P*	SMD
	(n=63)	(n=189)		
Age (years)	55.00 (48.50, 61.00)	54.00 (48.00, 61.00)	0.531	0.089
Gender				
Male	47 (74.60%)	144 (76.19%)	0.932	0.037
Female	16 (25.40%)	45 (23.81%)		
Child Pugh				
A	55 (87.30%)	168 (88.89%)	0.909	0.049
B	8 (12.70%)	21 (11.11%)		
ECOG PS				
0	56 (88.89%)	165 (87.30%)	0.912	0.049
1	7 (11.11%)	24 (12.70%)		
CNLC stage				
IIb	42 (66.67%)	132 (69.84%)	0.753	0.068
IIIa	21 (33.33%)	57 (30.16%)		

### 3.2 Survival outcomes in the overall and subgroup populations

Calculated from the time of meeting resectability criteria, the CMT group yielded an mOS of 24.3 months and an mPFS of 10.6 months. SR was significantly associated with prolonged survival, with the mOS and mPFS reaching 32.1 and 15.3 months, respectively. The 12-, 24-, and 36-month PFS rates for the CMT group were 44.5%, 22.0%, and 19.9%, compared to 66.7%, 36.2%, and 29.0% in the SR group (**Table 2**). Similarly, the SR group’s OS rates at 12-, 24-, and 36-months (98.4%, 81.3%, 44.6%) consistently outperformed the CMT group (88.1%, 50.8%, 10.3%). Kaplan-Meier analyses confirmed these significant advantages in both OS (χ2 = 16.59, P < 0.0001; shown in **Figure 2A**) and PFS (χ2 = 8.24, P = 0.0041; shown in **Figure 2B**).

**Table 2:** Comparison of mOS, mPFS, and 12-, 24-, and 36-month OS and PFS rates between the SR and CMT groups.

Parameters	SR	95%CI	CMT	*95%CI*	*χ^2^*	*P*
mOS (months)	32.1	29.9-39.4	24.3	21.6-27.5		
mPFS (months)	15.3	12.4-29.6	10.6	9.7-12.5		
Overall survival probability						
12-month	98.4%	95.4%-100.0%	88.1%	83.6%-92.9%	16.59	<0.001
24-month	81.3%	71.9%-91.9%	50.8%	43.5%-59.2%		
36-month	44.6%	32.0%-62.2%	10.3%	4.1%-25.6%		
Progression-free survival probability						
12-month	66.7%	56.0%-79.4%	44.5%	37.9%-52.2%	8.24	0.004
24-month	36.2%	26.0%-50.3%	22.0%	16.5%-29.5%		
36-month	29.0%	18.4%-45.7%	19.9%	14.4%-27.5%		

**Figure 2:**
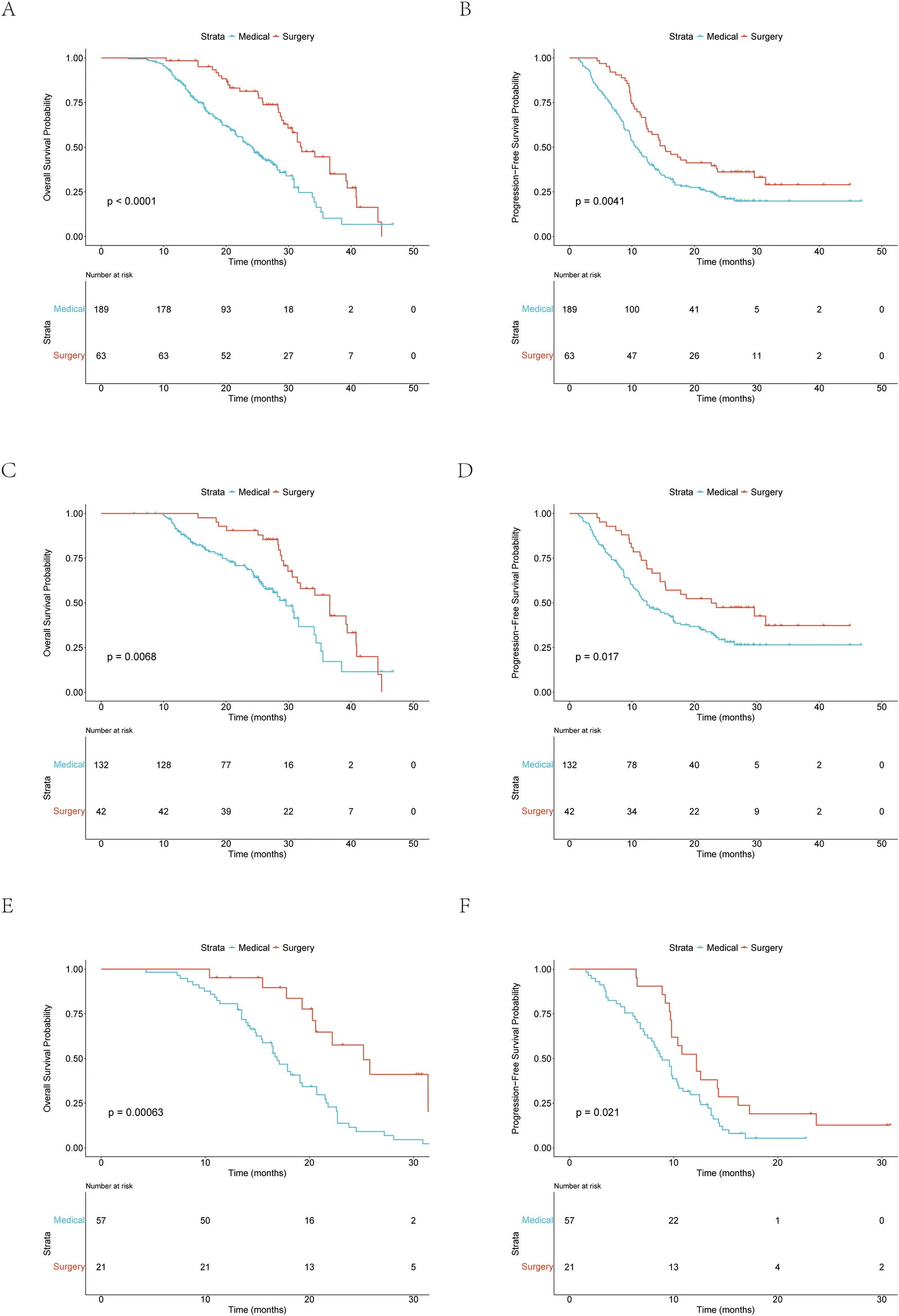
Kaplan-Meier survival curves comparing Surgical Resection (SR) and Continued Medical Treatment (CMT) after propensity score matching. **(A)** Overall survival (OS) in the matched overall cohort. **(B)** Progression-free survival (PFS) in the matched overall cohort. **(C)** OS in the CNLC stage IIb subgroup. **(D)** PFS in the CNLC stage IIb subgroup. **(E)** OS in the CNLC stage IIIa subgroup. **(F)** PFS in the CNLC stage IIIa subgroup. P-values were calculated using the log-rank test, demonstrating significant survival advantages for the SR group across all subsets.

These survival benefits remained consistent across subgroups. Baseline characteristics were well-balanced post-matching in both CNLC Stage IIb and IIIa groups (P > 0.05, SMD < 0.1, **Table 3**). Among the 174 CNLC stage IIb patients, the SR group maintained significant superiority in both OS (χ2 = 7.32, P = 0.0068; shown in **Figure 2C**) and PFS (χ2 = 5.72, P = 0.017; shown in **Figure 2D**). Correspondingly, among the 78 CNLC stage IIIa patients, the SR group also demonstrated significantly better OS (χ2 = 11.69, P = 0.00063; shown in **Figure 2E**) and PFS (χ2 = 5.36, P = 0.021; shown in **Figure 2F**) than the CMT group.

**Table 3:** Baseline characteristics of the SR and CMT groups after PSM: subgroup analysis by CNLC stage IIb and IIIa.

Variables	SR	CMT	*P*	SMD
CNLC-IIb (n=174)				
Age(years)	54.50 (48.00, 61.75)	53.00 (47.75, 60.00)	0.566	0.098
Gender				
Male	31 (73.81%)	100 (75.76%)	0.960	0.045
Female	11 (26.19%)	32 (24.24%%)		
Child Pugh				
A	37 (88.10%)	118 (89.39%)	1.000	0.041
B	5 (11.90%)	14 (10.61%)		
ECOG PS				
0	37 (88.10%)	115 (87.12%)	1.000	0.030
1	5 (11.90%)	17 (12.88%)		
CNLC-IIIa (n=78)				
Age(years)	55 (52, 60)	55 (49, 61)	0.828	0.056
Gender				
Male	16 (76.19%)	44 (77.19%)	1.000	0.024
Female	5 (23.81%)	13 (22.81%)		
Child Pugh				
A	18 (85.71%)	50 (87.72%)	1.000	0.059
B	3 (14.29%)	7 (12.28%)		
ECOG PS				
0	19 (90.48%)	50 (87.72%)	1.000	0.089
1	2 (9.52%)	7 (12.28%)		

### 3.3 Construction and validation of the prognostic model for the CMT group

In the CMT cohort, univariate and multivariate Cox regressions identified CNLC stage (HR = 4.332, 95% CI: 2.870–6.539, P < 0.001) and baseline AFP levels (AFP_1; HR = 1.001, 95% CI: 1.001–1.001, P < 0.001) as independent risk factors for mortality (**Table 4**). A corresponding prognostic nomogram was established (shown in **Figure 3A**), successfully satisfying the proportional hazards assumption (P = 0.085; shown in **Figure 3B**). Time-dependent ROC curves for the training cohort yielded excellent AUCs of 0.836, 0.874, and 0.855 at 12-, 24-, and 36-months, respectively (shown in **Figure 3C**). Calibration plots indicated strong agreement between predicted and observed probabilities at 12- (MAE = 0.012) and 24-months (MAE = 0.023), while estimations at 36-months were limited by lower event densities (shown in **Figure 3D**). The model exhibited robust discriminative capability in the internal validation cohort (12-, 24-, 36-month AUC = 0.815, 0.734, 0.778; shown in **Figure 3E**). DCA confirmed that all the models demonstrated greater net benefit than the “treat all” strategy across the majority of the threshold probability range, validating its utility in stratifying long-term risk for patients undergoing continued medical management (shown in **Figure 3F**).

**Table 4:** Results of univariate and multivariate Cox regression analyses in the CMT group.

Variables	CMT	Univariate analysis		Multivariate analysis	
		*HR (95%CI)*	*P*	*HR (95%CI)*	*P*
Age(years)	54.00 (48.00, 61.00)	0.984 (0.957, 1.011)	0.244		
Gender					
Male	144 (76.19%)	1.063 (0.678, 1.667)	0.790		
Female	45 (23.81%)				
Child Pugh					
A	168 (88.89%)	0.998 (0.567, 1.758)	0.995		
B	21 (11.11%)				
ECOG PS					
0	165 (87.30%)	0.557 (0.298, 1.042)	0.067		
1	24 (12.70%)				
CNLC Stage					
IIb	132 (69.84%)	4.171 (2.786, 6.246)	<0.001	4.332 (2.870, 6.539)	<0.001
IIIa	57 (30.16%)				
NEU_1(10^9/L)	3.06 (2.02, 4.23)	0.985 (0.903, 1.075)	0.737		
LYM_1(10^9/L)	1.01 (0.70, 1.31)	1.009 (0.699, 1.457)	0.962		
NLR_1	3.11 (2.14, 4.23)	0.990 (0.922, 1.064)	0.795		
PLT_1(10^9/L)	113.00 (76.00, 181.00)	1.000 (0.997, 1.002)	0.952		
PLR_1	117.36 (81.97, 163.64)	1.000 (0.997, 1.003)	0.996		
TB_1(μmol/L)	18.70 (14.60, 27.50)	0.998 (0.986, 1.010)	0.766		
ALB_1(g/L)	36.38 ± 5.40	1.007 (0.971, 1.044)	0.709		
INR_1	1.01 (0.96, 1.12)	1.927 (0.562, 6.609)	0.297		
AFP_1(ng/mL)	153.40 (38.31, 574.60)	1.001 (1.001, 1.001)	<0.001	1.001 (1.001, 1.001)	<0.001
Interval to Successful Conversion Therapy(months)	3.00 (3.00, 5.00)	0.968 (0.855, 1.096)	0.605		
NEU_2(10^9/L)	2.61 (1.84, 4.07)	0.892 (0.790, 1.007)	0.065		
LYM_2(10^9/L)	1.09 (0.77, 1.51)	0.999 (0.675, 1.481)	0.998		
NLR_2	2.56 (1.65, 3.83)	0.948 (0.865, 1.040)	0.259		
PLT_2(10^9/L)	99.00 (70.00, 153.00)	0.999 (0.996, 1.002)	0.419		
PLR_2	95.71 (64.90, 138.80)	0.999 (0.997, 1.002)	0.555		
TB_2(μmol/L)	19.00 (14.50, 25.50)	0.994 (0.978, 1.010)	0.460		
ALB_2(g/L)	37.60 (34.40, 41.10)	0.994 (0.960, 1.029)	0.723		
INR_2	1.01 (0.93, 1.12)	1.166 (0.471, 2.888)	0.740		
AFP_2(ng/mL)	98.40 (16.56, 309.90)	1.001 (1.001, 1.001)	<0.001	1.000 (1.000, 1.000)	0.907
TRAEs					
ND	60	0.981 (0.754, 1.278)	0.889		
1~2	119				
3	10				

*NEU, neutrophil count; LYM, lymphocyte count; NLR, neutrophil-to-lymphocyte ratio; PLT, platelet count; PLR, platelet-to-lymphocyte ratio; TB, total bilirubin; ALB, albumin; INR, international normalized ratio; AFP, alpha-fetoprotein;ND, not detected.

**Figure 3:**
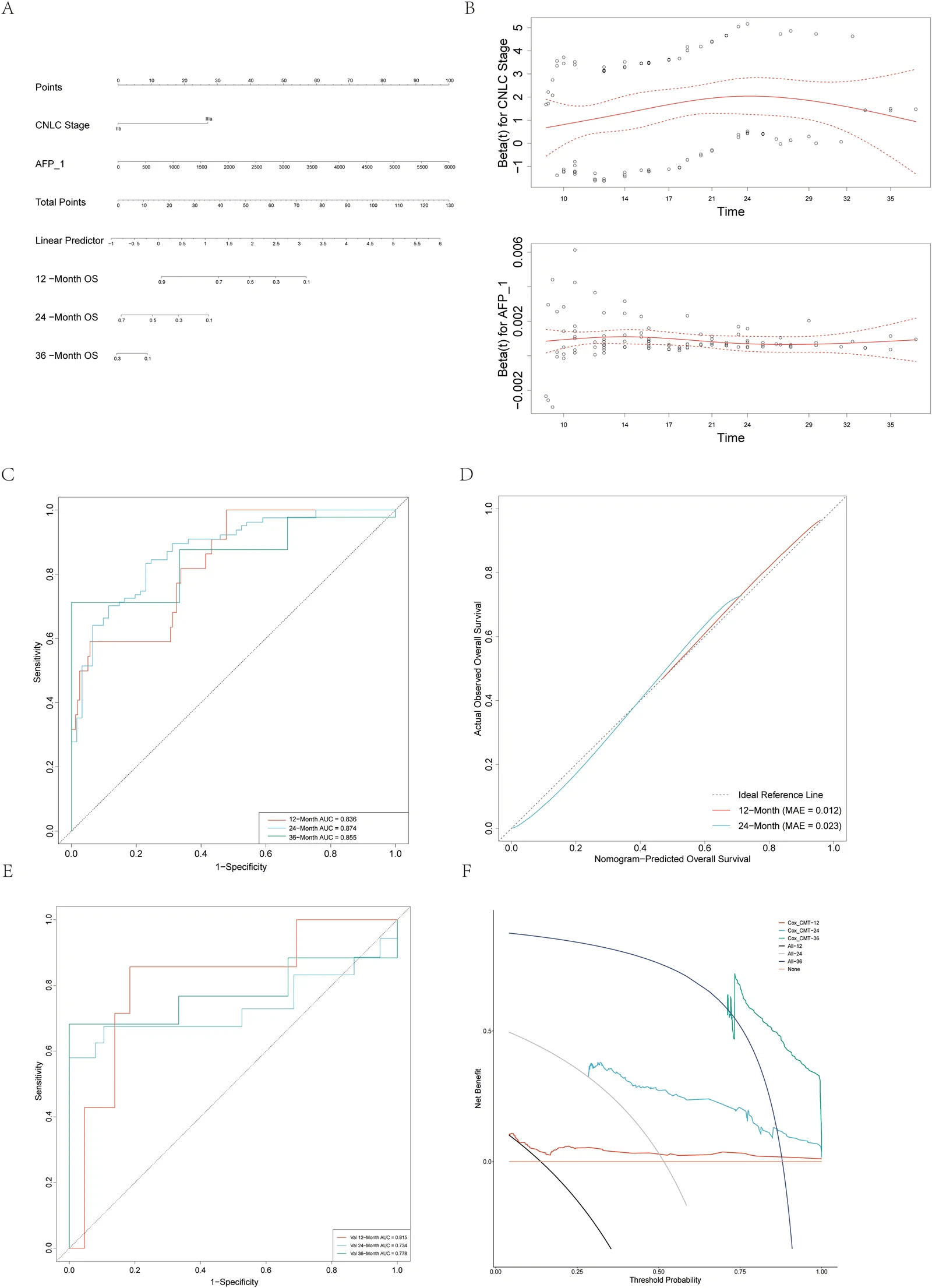
Construction and validation of the prognostic model for patients receiving Continued Medical Treatment (CMT). **(A)** A prognostic nomogram incorporating CNLC stage and baseline AFP to predict 12-, 24-, and 36-month OS. **(B)** Proportional hazards assumption testing for the CMT model variables. **(C)** Time-dependent receiver operating characteristic (ROC) curves in the training cohort. **(D)** Calibration plots comparing predicted and observed survival probabilities at 12, 24, and 36 months in the training cohort. **(E)** Time-dependent ROC curves demonstrating model discrimination in the internal validation cohort. **(F)** Decision curve analysis (DCA) illustrating the clinical net benefit of the 36-month survival prediction model across varying threshold probabilities.

### 3.4 Prognostic model and exploratory analysis of surgical timing for the SR group

For the 63 matched patients receiving surgery (with 36 death events observed by the end of the follow-up period), multivariate analysis identified three independent prognostic variables (**Table 5**): CNLC stage (HR = 3.116, 95% CI: 1.261–7.703, P = 0.014), the ratio of preoperative AFP to resectability baseline AFP (HR = 3.151, 95% CI: 1.329–7.472, P = 0.009), and the interval from meeting resectability criteria to surgery (HR = 1.012, 95% CI: 1.004–1.020, P = 0.004). The formulated nomogram model (shown in **Figure 4A**) met the proportional hazards assumption (P = 0.063; shown in **Figure 4B**). Importantly, RCS analysis revealed a distinct non-linear relationship between surgical delay and mortality risk. Using the median interval (90 days) as the reference point (HR = 1.0), the mortality risk remained relatively stable if surgery was performed within 90 days but increased steadily thereafter, underscoring the detrimental impact of delaying surgery after successful conversion (shown in **Figure 4C**). This SR model demonstrated robust predictive efficacy. In the training set, the 12-, 24-, and 36-month AUCs were 0.950, 0.934, and 0.794, respectively, accompanied by low mean absolute errors (shown in **Figures 4D, E**). The results were similarly solid in the internal validation cohort (shown in **Figure 4F**). DCA reaffirmed the high net clinical benefit of this tailored model (shown in **Figure 4G**). To directly inform clinical decisions, Kaplan-Meier analyses clarified that an AFP ratio > 1 strongly predicted worse OS compared to a ratio ≤ 1 (χ2 = 21.31, P < 0.0001; shown in **Figure 4H**), and an interval to surgery ≤ 90 days was significantly associated with superior OS compared to prolonged waiting times > 90 days (χ2 = 4.78, P = 0.029; shown in **Figure 4I**). Therefore, our exploratory analysis suggests that a potential surgical strategy involves executing definitive resection within 90 days of achieving resectability, stringently ensuring that the preoperative AFP has not rebounded beyond its baseline level at the time of conversion success.

**Table 5:** Results of univariate and multivariate Cox regression analyses in the SR group.

Variables	SR	Univariate analysis		Multivariate analysis	
		*HR (95%CI)*	*P*	*HR (95%CI)*	*P*
Age(years)	55.00 (48.50, 61.00)	0.982 (0.942, 1.024)	0.400		
Gender					
male	47 (74.60%)	1.034 (0.481, 2.223)	0.931		
female	16 (25.40%)				
Child Pugh					
A	55 (87.30%)	0.967 (0.365, 2.564)	0.947		
B	8 (12.70%)				
ECOG PS					
0	56 (88.89%)	0.302 (0.072, 1.269)	0.102		
1	7 (11.11%)				
CNLC Stage					
IIb	42 (66.67%)	3.446 (1.539, 7.712)	0.003	3.116 (1.261, 7.703)	0.014
IIIa	21 (33.33%)				
NEU_1(10^9/L)	4.35 (2.98, 5.67)	1.000 (0.898, 1.114)	0.998		
LYM_1(10^9/L)	1.04 (0.84, 1.38)	0.722 (0.307, 1.696)	0.455		
NLR_1	3.66 (2.64, 5.89)	0.986 (0.925, 1.050)	0.655		
PLT_1(10^9/L)	162.00 (111.50, 221.00)	0.997 (0.993, 1.001)	0.103		
PLR_1	156.41 (100.22, 223.67)	0.998 (0.994, 1.002)	0.266		
TB_1(μmol/L)	22.90 (15.75, 32.80)	1.002 (0.967, 1.037)	0.923		
ALB_1(g/L)	37.95 ± 5.21	0.940 (0.879, 1.005)	0.069		
INR_1	1.00 (0.93, 1.08)	1.312 (0.028, 61.089)	0.890		
AFP_1(ng/mL)	31.60 (17.38, 122.00)	1.000 (1.000, 1.000)	0.553		
Interval to Successful Conversion Therapy(months)	4.00 (3.00, 6.00)	1.006 (0.877, 1.155)	0.929		
NEU_2(10^9/L)	4.34 ± 1.87	1.007 (0.833, 1.218)	0.940		
LYM_2(10^9/L)	1.12 (0.85, 1.39)	1.071 (0.883, 1.298)	0.486		
NLR_2	3.91 (2.18, 5.72)	1.006 (0.876, 1.156)	0.934		
PLT_2(10^9/L)	166.51 ± 69.65	0.996 (0.992, 1.001)	0.154		
PLR_2	142.42 (88.48, 181.26)	0.999 (0.995, 1.002)	0.462		
TB_2(μmol/L)	32.30 (18.85, 45.30)	0.993 (0.981, 1.006)	0.306		
ALB_2(g/L)	38.90 (34.30, 43.05)	1.026 (0.960, 1.098)	0.447		
INR_2	1.03 (0.94, 1.14)	2.036 (0.140, 29.518)	0.602		
AFP_2(ng/mL)	22.84 (8.73, 89.69)	1.000 (1.000, 1.001)	0.388		
NEU_3(10^9/L)	4.22 ± 1.65	1.216 (0.983, 1.505)	0.071		
LYM_3(10^9/L)	0.99 (0.75, 1.32)	1.431 (0.571, 3.590)	0.445		
NLR_3	3.95 (2.69, 6.12)	1.123 (0.969, 1.301)	0.123		
PLT_3(10^9/L)	167.95 ± 69.68	0.998 (0.994, 1.003)	0.460		
PLR_3	157.02 (101.40, 256.35)	0.999 (0.996, 1.002)	0.507		
TB_3(μmol/L)	29.40 (20.05, 38.60)	0.996 (0.978, 1.015)	0.682		
ALB_3(g/L)	38.70 (37.05, 42.20)	1.030 (0.948, 1.118)	0.483		
INR_3	1.00 ± 0.06	5.897 (0.017, 2006.324)	0.551		
AFP_3(ng/mL)	20.64 (8.56, 94.44)	1.000 (1.000, 1.000)	0.448		
AFP Ratio Status					
0	41 (65.08%)	5.341 (2.440, 11.691)	<0.001	3.151 (1.329, 7.472)	0.009
1	22 (34.92%)				
TRAE					
ND	35 (55.56%)	1.403 (0.890, 2.214)	0.145		
1~2	26 (41.27%)				
3	2 (3.17%)				
Interval to Operation(days)	90.00 (43.50, 122.50)	1.012 (1.005, 1.020)	0.001	1.012 (1.004, 1.020)	0.004

*NEU, neutrophil count; LYM, lymphocyte count; NLR, neutrophil-to-lymphocyte ratio; PLT, platelet count; PLR, platelet-to-lymphocyte ratio; TB, total bilirubin; ALB, albumin; INR, international normalized ratio; AFP, alpha-fetoprotein;ND, not detected.

**Figure 4:**
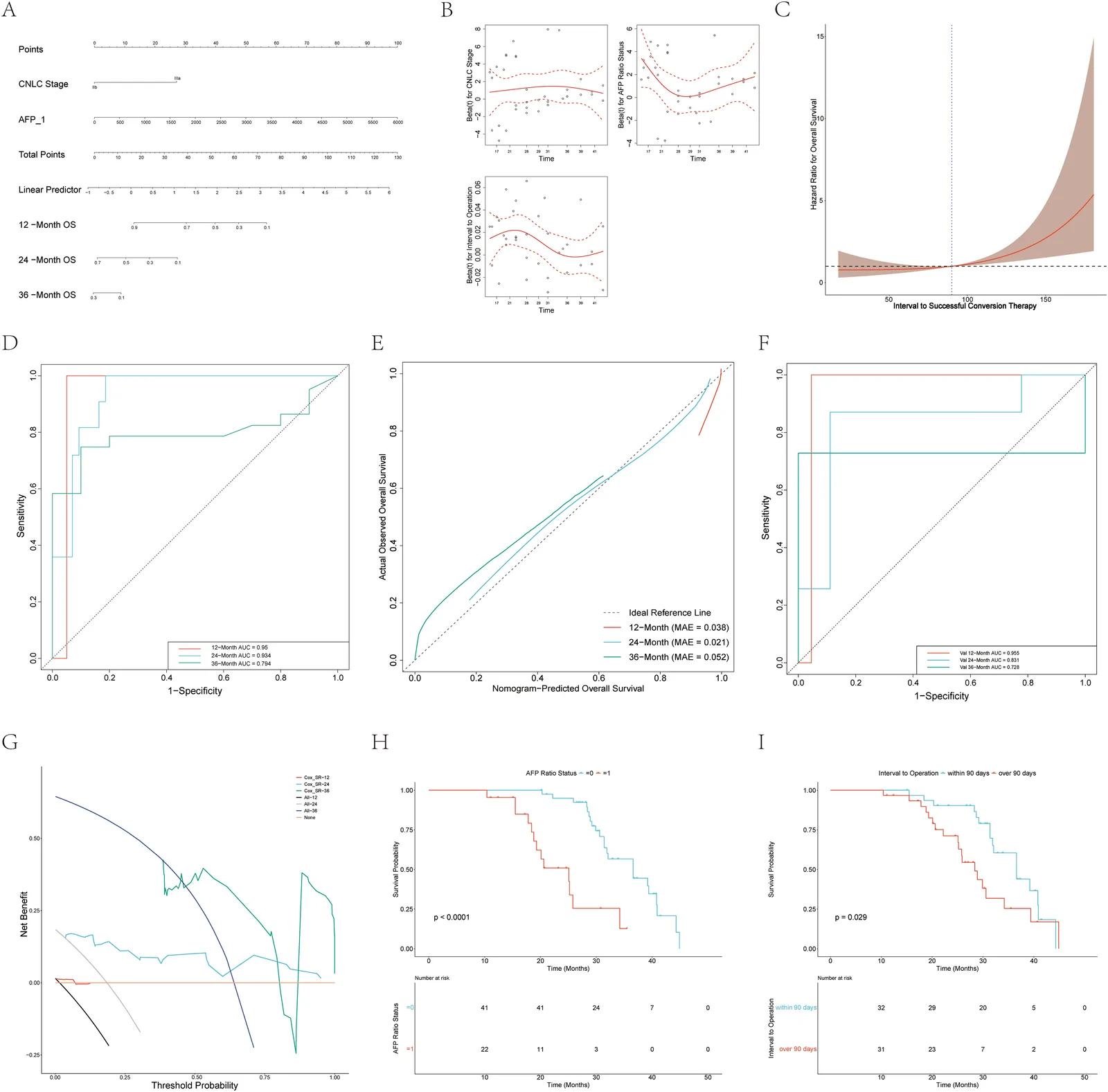
Prognostic nomogram construction and exploration of surgical timing for patients undergoing Surgical Resection (SR). **(A)** A prognostic nomogram incorporating CNLC stage, the interval to operation, and AFP ratio status to predict 12-, 24-, and 36-month OS. **(B)** Proportional hazards assumption testing for the SR model variables. **(C)** Restricted cubic spline (RCS) analysis revealing a non-linear relationship between the interval from meeting resectability criteria to surgery and the risk of mortality. A clear inflection point is observed at 90 days. **(D)** Time-dependent ROC curves in the training cohort. **(E)** Mean absolute error (MAE) of the predictive model over time. **(F)** Time-dependent ROC curves in the internal validation cohort. **(G)** Decision curve analysis (DCA) of the SR nomogram. **(H)** Kaplan-Meier OS curves stratified by preoperative AFP/baseline AFP ratio (≤1 vs. >1). **(I)** Kaplan-Meier OS curves stratified by the surgical waiting interval (≤90 days vs. >90 days), suggesting a potential survival benefit of early surgical intervention within 90 days.

To simplify the practical use of our model in routine clinical workflows, we implemented an open-access web application (https://jianqiu.shinyapps.io/hcc-predictor/). Clinicians can seamlessly switch between SR and CMT treatment arms via the drop-down menu. Upon entering a patient’s baseline clinical variables and desired time point (e.g., 12, 36, or 60 months), the app instantly calculates the estimated overall survival rate and displays the individualized survival trajectory.

## 4 Discussion

Conversion therapy offers new hope for patients with CNLC stage IIb/IIIa HCC. This study confirms that for patients meeting tumor resectability criteria, subsequent surgical resection is associated with significantly superior OS and PFS compared to maintaining original medical therapy. Furthermore, we developed high-precision prognostic nomograms for both clinical scenarios and, for the first time, identified a potential 90-day time window for post-conversion surgical intervention as an exploratory finding.

The SR group exhibited a profound survival advantage (OS, χ2 = 16.59, P < 0.0001). Biologically, surgery thoroughly eradicates residual macroscopic tumor burden and potential drug-resistant clones that may evolve under prolonged pharmacological pressure (7, 30, 31). Notably, the incidence of grade 1–2 TRAEs was significantly lower in the SR group (41.27% vs. 62.96%), likely because definitive surgery allows for the safe de-escalation or suspension of systemic agents, thereby mitigating cumulative toxicity (12, 32, 33).

However, the proportion of patients undergoing surgery after meeting resectability criteria in actual clinical practice remained low. In prior studies, Jia-Yi Wu et al. demonstrated that among 62 patients enrolled in a triple-modality regimen of transarterial chemoembolization (TACE), lenvatinib, and Anti-PD-1 Antibodies, 33 patients (53.2%) reached the standard of conversion to resectable HCC and 29 patients underwent resection (24). Separately, Shichun Lu et al. reported on 120 treatment-naive patients with unresectable hepatocellular carcinoma treated with sintilimab plus lenvatinib, achieving a conversion success rate of 56% (67/120). Of these 67 patients who attained resectability, 60 ultimately proceeded to definitive surgery (34). In our study, only 90 patients underwent surgery, representing 10.47% of the total 860 patients receiving conversion therapy and 24.79% of the 363 patients who were retrospectively confirmed to have met the resectability criteria. This attrition can be attributed to several factors (1): emergence of new extrahepatic/vascular progression or significant deterioration of FLR/ICG during the waiting period (2); patient refusal due to financial constraints, poor compliance, fear of surgery, or uncertainty regarding surgical benefits; and (3) in real-world practice, a lack of timely surgical evaluation during long-term follow-up in medical oncology or infectious disease departments. In our cohort, although 363 patients biologically met the criteria, only 48.48% (176/363) were dynamically evaluated for surgical indications during their actual clinical care. This highlights the critical necessity of establishing a liver surgery-centric, prospective Multidisciplinary Team (MDT) in routine clinical practice to ensure that CNLC stage IIb/IIIa patients who achieve disease control receive timely surgical evaluation and intervention (12, 35).

Several limitations of this study should be acknowledged. First, an inherent immortal time bias may exist, as patients in the SR group had to survive and remain progression-free until undergoing surgery. Although the baseline was strictly defined from the date resectability was achieved, future studies using time-dependent Cox models or landmark analyses are warranted to confirm the surgical survival advantage. Second, the surgical prognostic model was developed from a limited matched cohort of 63 patients, carrying a potential risk of overfitting. While resampling evaluation via 1, 000 bootstrap replicates confirmed the model’s robustness, the SR cohort in the internal validation set remained relatively small. Furthermore, although the proposed nomogram demonstrated satisfactory discrimination, well-fitted calibration, and potential clinical utility, a notable limitation is the lack of true external validation (36). Larger multicenter cohorts are required for further validation. Third, the single-center, retrospective design leaves room for potential residual confounding and selection bias. Although PSM achieved an optimal baseline balance (all SMDs < 0.1), residual confounding by indication remains likely, as patients selected for surgery may differ inherently in tumor biology, response durability, fitness, technical operability, and clinician/patient preferences; this should be acknowledged when interpreting the apparent survival association (37). Furthermore, being a single-center Chinese cohort treated with a specific lenvatinib–PD-1–TACE regimen and assessed using CNLC-based resectability criteria, the generalizability of our findings is limited. Given that conversion therapy is evolving and resectability criteria as well as underlying HCC etiologies vary between regions, further validation across diverse populations is necessary (38).​ Finally, the relatively short median follow-up led to a reduced density of endpoint events at 36 months, limiting the precision of long-term survival estimations. Large-scale, multicenter prospective trials are therefore necessary to validate these findings.

A major innovative finding of this study is the exploration of a potential surgical timing. Using RCS and Kaplan-Meier analyses, we demonstrated that delaying surgery beyond 90 days after meeting resectability criteria is significantly associated with an increased mortality risk. However, this proposed 90-day window should be interpreted cautiously. The 90-day cut-off closely relates to the observed median time to surgery in our cohort, and this retrospective association does not establish a definitive causal threshold. Furthermore, previous evidence examining treatment delays in HCC has shown considerable heterogeneity, without establishing 90 days as a universal adverse threshold (39). Prolonged exposure to TKIs and ICIs may exacerbate hepatic fibrosis, damage vascular walls, and induce immune cell exhaustion, complicating surgery and impairing postoperative functional recovery. Additionally, an elevated preoperative AFP ratio (> 1) serves as an early indicator of tumor immune escape.

The developed prognostic models exhibited excellent discrimination and calibration. While CNLC stage and baseline AFP were primary predictors in the CMT group, temporal variables (surgical interval) and dynamic biomarkers (AFP ratio) dominated the SR model, underscoring the critical importance of “time variables” in the context of conversion therapy.

## 5 Conclusion

For CNLC stage IIb/IIIa HCC patients meeting tumor resectability criteria, surgical resection is associated with significantly superior survival and safety profiles compared to continuing original medical treatments. To maximize survival benefits, surgical resection within 90 days of meeting resectability criteria could be considered as an exploratory strategy, provided that preoperative AFP levels do not rebound.

## Data Availability

The raw data supporting the conclusions of this article will be made available by the authors, without undue reservation.
